# PD06 New French Recommendations For The Coverage Of Connected Systems For Diabetes Care

**DOI:** 10.1017/S026646232510144X

**Published:** 2025-12-29

**Authors:** Estelle Piotto, Emmanuelle Fouteau, Coralie Jarraud, Audrey Nganbou, Hubert Galmiche, Floriane Pelon

## Abstract

**Introduction:**

Since 2016, the emergence of continuous glucose monitors and closed-loop systems has significantly changed the way diabetes is managed. They have reduced the number of finger pricks and optimized insulin therapy by automatically adjusting insulin doses. In this evolving context, the Haute Autorité de santé (HAS) reassessed the national coverage conditions of three categories of medical devices for self-monitoring and automated insulin therapy management.

**Methods:**

The assessment method was based on analysis of data from: (i) a systematic review of the scientific literature; (ii) proposals from concerned stakeholders (patient associations, professional organizations, manufacturers, service providers, public health institutions); and (iii) information from international health technology assessment agencies. The HAS assessment report and the new proposed reimbursement nomenclature were validated in July 2024 by the HAS committee, which appraises medical devices with respect to their reimbursement by the French health insurance scheme.

**Results:**

The main changes to the coverage conditions included harmonized and extended indications for each category of device, which involved removing the previous requirement of six months of insulin pump use and the metabolic criterion (hemoglobin A1c level). The prescribing conditions were also updated, allowing prescriptions from public or private hospital or non-hospital based centers. Concerning relative clinical effectiveness, HAS concluded that there was no clinical added benefit between the devices in each category. The evaluation of specific clinical data for each device carried out by HAS remains necessary before considering coverage in France, and post-registration studies are still required for closed-loop systems.

**Conclusions:**

The new HAS coverage recommendations for the three categories of connected medical devices for managing diabetes offer greater clarity and improve patient care. More patients will benefit from these systems due to the extended indications. Moreover, the post-registration studies required by HAS for each closed-loop system will allow the long-term evaluation of these systems in France.

